# Enhancement of Antibacterial Properties, Surface Morphology and In Vitro Bioactivity of Hydroxyapatite-Zinc Oxide Nanocomposite Coating by Electrophoretic Deposition Technique

**DOI:** 10.3390/bioengineering10060693

**Published:** 2023-06-07

**Authors:** Waseem Akram, Rumaisa Zahid, Raja Muhammad Usama, Salman Ali AlQahtani, Mostafa Dahshan, Muhammad Abdul Basit, Muhammad Yasir

**Affiliations:** 1 Department of Mechanical Engineering, Faculty of Engineering & Technology, International Islamic University, Islamabad 44000, Pakistan; waseem.akram@iiu.edu.pk; 2 Department of Materials Science & Engineering, Institute of Space Technology, Islamabad 44000, Pakistan; 3 Department of Computer Engineering, College of Computer and Information Sciences, King Saud University, P.O. Box 51178, Riyadh 11543, Saudi Arabia; 4 School of Computing, Mathematics and Engineering, Charles Sturt University, Panorama Avenue, Bathurst, NSW 2795, Australia

**Keywords:** electrophoretic deposition, invitro study, hydroxyapatite, nanocomposites, surface morphology, zinc oxide

## Abstract

To develop medical-grade stainless-steel 316L implants that are biocompatible, non-toxic and antibacterial, such implants need to be coated with biomaterials to meet the current demanding properties of biomedical materials. Hydroxyapatite (HA) is commonly used as a bone implant coating due to its excellent biocompatible properties. Zinc oxide (ZnO) nanoparticles are added to HA to increase its antibacterial and cohesion properties. The specimens were made of a stainless-steel grade 316 substrate coated with HA-ZnO using the electrophoretic deposition technique (EPD), and were subsequently characterized using scanning electron microscopy (SEM), energy dispersive X-ray (EDX), stylus profilometry, electrochemical corrosion testing and Fourier transform infrared (FTIR) spectroscopy. Additionally, cross-hatch tests, cell viability assays, antibacterial assessment and in vitro activity tests in simulated body fluid (SBF) were performed. The results showed that the HA-ZnO coating was uniform and resistant to corrosion in an acceptable range. FTIR confirmed the presence of HA-ZnO compositions, and the in vitro response and adhesion were in accordance with standard requirements for biomedical materials. Cell viability confirmed the viability of cells in an acceptable range (>70%). In addition, the antibacterial activity of ZnO was confirmed on Staphylococcus aureus. Thus, the HA-ZnO samples are recommended for biomedical applications.

## 1. Introduction

Bone implants have been important in the treatment of damaged and fractured bones for several decades. Several materials such as titanium and its alloys, stainless steel, cobalt and magnesium are used as bone implants [1].

Organic and inorganic coatings on implants are used to achieve biocompatibility, inflammation resistance and to exhibit antibacterial behavior. Out of the vast range of available coatings, hydroxyapatite (HA) is one of the preferred inorganic coatings due to its excellent biomedical properties, including its bioactivity and biocompatibility [2]. It further helps in regenerative purposes and enhances bone healing [3]. Among several methods of depositing HA coatings on bone implants, the plasma spray coating technique was used to deposit particles of different particle sizes [4]. It was observed that the morphology and crystallinity of the HA film on a substrate depends on the particle size of HA powders [5]. A more crystalline structure of HA films was observed using amorphous powders through combustion flame spraying [6]. S.W.K. Kweh [7] observed that the fracture toughness and the elastic modulus of the HA coatings is enhanced through plasma spraying [8]. The particle size of the HA used as feedstock has a significant effect on the mechanical properties of HA films The electrophoretic deposition technique is capable of producing coatings that possess a range of functionalities, including bioactivity, antibacterial action and biocompatibility, thus making them multifunctional [9]. M. Sabzi et al. coated HA-ZnO on a NiTi shape memory alloy of 135 µm thickness via EPD and concluded that the coating was uniform, crack-free, biologically active and highly resistant to corrosion [10]. A. Kand et al. deposited HA on AZ31 via EPD and observed that the degradation of the AZ31 alloy became passive due to the HA coating and biological apatite sediments formed upon immersion in SBF [11].

To further enhance the anti-bacterial, bone regeneration and cohesion properties of bone implants, zinc oxide (ZnO) nanopowder is an excellent choice for implant coatings [12]. The United States Federal Drug Administration (21CFR182.8991) has classified ZnO as generally recognized as safe (GRAS) [13]. ZnO powder is often mixed with other coating materials to enhance their anti-inflammatory, antibacterial and cohesive properties. However, limited research studies are available on the characterization of such ZnO-reinforced coatings. Puneet Bansal et al. [14] used the plasma spray technique to characterize HA-ZnO coatings on an AZ31 Mg alloy. They observed that the addition of ZnO improved the surface properties and corrosion resistance of the coatings. Jingan Li et al. investigated that the alloy of Mg-xLi-Zn is highly recommended for bone implants due to the development of mechanical properties.

The addition of ZnO in HA was reported to improve cell viability and antibacterial properties, and no cytotoxic effect on the structural organization of the cells was observed [15].

Kummara Madhusudana Rao et al. [16] conducted an investigation on the preparation of hyaluronic acid–zinc oxide hydrogels using a one-pot method. These hydrogels exhibited excellent antibacterial properties and were found to be effective hemostatic agents. Based on these findings, the researchers suggest that hydrogels are potential candidates for their use as wound dressing materials. According to Baikere Maimaiti [17], a stable nanocoating of ZnO-doped hydroxyapatite on titanium can be beneficial for both anti-infection and osteogenic purposes.

Puneet Bansal [18] investigated Ti_13_Nb_13_Zr titanium alloy with plasma-sprayed HA-ZnO coatings for biomedical applications. They observed that the addition of ZnO in HA improved hardness and antibacterial properties. Jie Zhou et al. [19] coated nano-HA-ZnO on biodegradable Mg-Zn-Ca via the hydrothermal method, and the results showed that the coated implant is more biologically active than the traditional metals. Jianghong Luo et al. [19] coated HA doped with multi-metal ions on a Ti substrate via the pulse electrochemical method and observed that the coated substrate was highly antibacterial and biologically friendly.

SS 316L [20,21] bone implants are more vulnerable to cytotoxicity compared to other implant types [22]. ZnO is specifically used to enhance the cohesion characteristics of the HA coatings applied to SS implants [23]. Moreover, HA-based zinc oxide nanocomposites possess similar mechanical properties to bones [24]. There are several reliable techniques that offer HA coatings, including radio frequency (RF) magnetron sputtering [25,26,27], plasma spraying [28,29,30], high-velocity oxy-fuel plasma (HVOF) coating [23], the sol–gel process [31] and the cold spray technique [32]. One major drawback of using these methods is the cost and maintenance of the equipment required and the lack of precise control over the coating process, which makes it very difficult to utilize RF sputtering [33], sol–gel [34] or HVOF [35] spraying techniques for intricate shapes such as dental implants. The electrophoretic deposition (EPD) method is a highly effective technique for coating metals, polymers, ceramics and composites with complex shapes and structures. In addition, this method enables the deposition of polymers, proteins and peptides at room temperature, without compromising their chemical composition [36]. Moreover, EPD has the ability to create multifunctional coatings that possess bioactive, antibacterial and biocompatible properties. EPD uses a simple setup that includes two electrodes, and it can be powered by either a direct current (DC) or alternating current (AC) [9] since, with the help of suspension, any sort of uniform coating can be achieved on the opposite electrode [37]. It has been observed that the deposition of HA-ZnO composite coatings on SS 316L via the EPD technique has not been carried out to date, as per the author’s knowledge.

The objective of this work is to investigate the effect of ZnO addition on HA coatings deposited on a SS 316L substrate via the EPD process and to analyze its resultant surface and anti-bacterial properties in depth via different characterization techniques and conduct in vitro testing in SBF medium.

## 2. Materials and Methods

### 2.1. Materials

ZnO powder (CAS 1314-13-2) with an average size of 600 nm and HA powder with an average particle size of 0.69 µm (Sigma Aldrich, St. Louis, MO, USA) were used, and coatings were deposited on SS 316 L substrate.

### 2.2. Suspension

The HA-ZnO coating on SS substrate using EPD technique was performed by making a suspension of the powders in ethanol. For the HA-ZnO composite powder suspension, 1.5 g of HA and 1 g of ZnO were added in 50 mL of pure ethanol. The suspension was sonicated for 8 h. These suspensions were mixed properly and placed on a 78HW-I thermostatic magnetic stirrer for 5 h before first use.

### 2.3. EPD Coating

**Figure 1:**
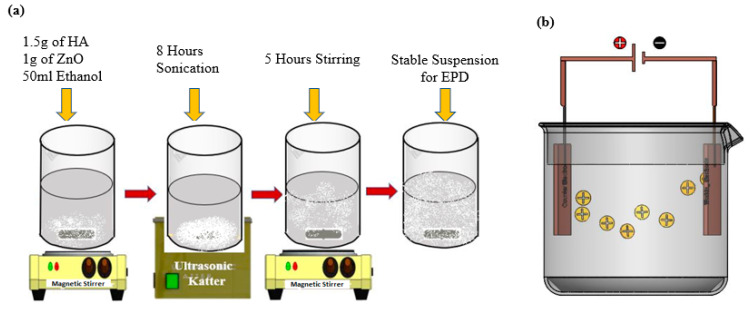
Schematic illustration of development of HA-ZnO-based coatings: (**a**) suspension preparation, (**b**) electrophoretic deposition (EPD) technique.

The parameters such as time and voltage for the experiments were optimized for the EPD process, as per previous reported literature [38]. A schematic diagram of suspension preparation and the EPD technique are shown in Figure 1a,b. To achieve coatings with good adhesion and surface properties, further EPD process parameters such as concentration, voltage and time were varied and optimized [39]. Next, 3 × 2 cm samples of stainless steel were hammered from edges and then cleaned with distilled water. The adhesion EPD coatings are sensitive to dust and debris, and special care was taken to protect them. EPD was set up by placing a stainless-steel anode and cathode in the suspensions one by one. SS substrates were coated by maintaining the following parameters 5 min, 20 V and 10 mm distance between electrodes. These suspensions were magnetically mixed after each coating in order to achieve excellent and uniform adhesion. pH was maintained for each suspension, i.e., 3.86 pH at 21 °C for HA-ZnO; pH was checked using a HANNA HI 2211 bench pH meter. The EPD setup and prepared samples are shown in Figures S1 and S2, respectively.

### 2.4. SEM Analysis

The morphology of the coating powder and coatings was examined using a TESCAN field emission scanning electron microscope (FESEM) (Brno, Czech Republic), with a resolution of 5 nm. Coated samples of size 1 × 1 cm were used to analyze the structure and morphology. The applied voltage during SEM was 20 kV. The elemental analysis was performed using an energy dispersive X-ray spectroscope (EDS, S-4700 with a Noran System 7, Hitachi, Tokyo, Japan).

### 2.5. Surface Roughness

Surface roughness of plays an important role in cell attachment and osteoblasting as ions release in physiological activity [40]. It was reported that good cell attachment was observed at a roughness value of 1–2 µm. Roughness of the HA-ZnO coatings was measured with stylus profilometer (TMR 360, Beijing, China). Samples were cleaned prior to testing. An average of three readings were measured to obtain the actual value.

### 2.6. Electrochemical Study

In order to assess the corrosion behavior of HA-ZnO coatings in SBF medium, it is necessary to perform electrochemical corrosion testing [41]. Initially, specimens were prepared by masking the desired surface area of the HA-ZnO-coated substrate. The SBF solution was prepared using ISO standards 10993-14 [42]. Electrochemical corrosion testing was carried out on the coated specimens using a potentiostat on Gamry setup (Farmwork 600, Louis Drive Warminster, PA, USA). A three-electrode configuration was utilized for this purpose, which includes the coated specimen as the working electrode, a reference electrode and a counter electrode. Samples were immersed in the SBF solution for a specific period of 24 h, which allowed them to stabilize. Potentiodynamic polarization curves were obtained by applying a potential range that covers the corrosion potential (Ecorr) and measures the corrosion current (Icorr) and corrosion potential (Ecorr) from the curve. Furthermore, the corrosion rate of the coated specimen in SBF solution was measured by applying a small-amplitude sinusoidal potential around the corrosion potential (Ecorr) and recording the corresponding current response. Finally, the results were drawn against the voltage potential 0.2 V and current density 1 × 10^−^^3^ A·cm^−^^2^. These results were compared with those obtained for uncoated specimens to determine the effectiveness or corrosion rate of the HA-ZnO coating.

### 2.7. FTIR

Fourier transform infrared (FTIR) spectroscopy (Shimadzu, Kyoto, Japan) setup was used for HA-ZnO powder, utilizing the parameters of transmittance and wave number. The samples were prepared using the standard technique [43], and FTIR measurements were conducted using a single-beam Fourier transform infrared spectrometer. For this purpose, the spectra of FTIR for the HA-ZnO-coated sample was taken in the spectral range from 4000 to 600 cm^−1^.

### 2.8. Adhesion Properties

The cross-hatch tape test was used to check the adhesion of the coating using ASTM D3359 and ISO 2409 standards [44]. Two cuts/scratches were made perpendicular to each other with an appropriate cutter, i.e., through the coating into the substrate to draw a lattice pattern. Pressure-sensitive adhesive tape (PSA, self-adhesive) was pasted on it with good contact and pulled back at a 180° angle. Each test was performed three times, and the average value was taken.

### 2.9. XRD

To investigate the phase composition of HA-ZnO, XRD analysis was carried out using an X-ray powder diffractometer (Malvern PANalytical Aeris, Cambridge, UK) using Cu Kα monochromatic radiation at a scan range of 5° to 90° with a stay time at each step of 4 sec and step size of 0.4. Phase identification was achieved by comparing the diffraction patterns using ICDD (JCPDS) standards.

### 2.10. In Vitro Study

The bioactivity of the coated samples (size 1 × 1 cm^2^) in the simulated body fluid were examined by weight loss and pH variation. To examine biocompatibility, the SBF (NaCl 7.996, NaHCO_3_ 0.35, KCl 0.224, K_2_HPO_4_·3H_2_O 0.228, MgCl_2_·6H_2_O 0.305, CaCl_2_ 0.278, Na_2_SO_4_ 0.071 and (CH_2_OH)_3_CNH_2_ 6.057) by concentration (g/L) was prepared using kokokubo [45] techniques, as the SBF is a solution utilized for in vitro bio-mineralization studies that imitates the chemical composition of human blood plasma. Its preparation protocol includes making a stock solution of salts, adding them to a volumetric flask, regulating the pH and sterilizing it by filtering [42]. In the current research, the biocompatibility of coated samples were examined for 24 h, 72 h, 120 h and 168 h in SBF solution, and the morphology was checked via SEM. For this purpose, the samples were taken out of the SBF and dried at room temperature to check the weight. In addition, weight gain/loss studies were also considered for HA-ZnO-coated samples in SBF for durations of 24 h, 72 h, 120 h and 168 h.

### 2.11. Cell Culture Study

The HA-ZnO coating’s in vitro biocompatibility was evaluated using human-derived stem cells. Stem cells are basically the origin of all kinds of cells. Cells were maintained at 37 °C in a humidified environment of 5% CO_2_ and grown in Dulbecco’s modified eagle’s medium (DMEM; Gibco, Billings, MT, USA) supplemented with 10 vol.% fetal bovine serum (FBS; Sigma-Aldrich, St. Louis, MO, USA) and 1 vol.% penicillin–streptomycin (Pen Strep; Sigma-Aldrich). The medium was removed after incubation, and cells were then cleaned with phosphate buffer saline (PBS; Gibco). The cells were separated from the flask wall using trypsin (Sigma-Aldrich, St. Louis, MO, USA) in the following steps. All materials were divided into 1 × 1 cm^2^ pieces and sterilized for 30 min using ultraviolet light to conduct cell tests. As a control, tissue culture plates (TCP) were utilized. In vitro cellular studies were carried out using an indirect assay (cells were in contact with the extracts of the scaffolds, which were obtained by immersing the coatings in DMEM).

### 2.12. Antibacterial Study

For the evaluation of antibacterial properties, the agar disc diffusion test was performed against Gram-positive (*Staphylococcus aureus*; *S. aureus*) and Gram-negative (*Escherichia coli*; *E. coli*) bacterial strains. Petri dishes were filled with 20 mL agar and LB medium inoculated with 20 μL *Staphylococcus (S. aureus)* and *Escherichia coli. E. coli* having an optical density of 0.015 (OD600) were spread on agar plates. Coated and uncoated samples were again placed in the agar plate for 24 h and incubated on 37 °C for antibacterial studies. Images of Petri dishes were captured to track the inhibition zone (if it existed). To monitor the inhibition zone in the antibacterial test, sterile filter paper discs were placed onto an agar plate, and an antimicrobial agent was added and incubated. Presence of an inhibition zone was checked and its size determined after incubation [46], and the results were reported.

## 3. Results and Discussion

### 3.1. Morphological and Compositional Analysis

**Figure 2:**
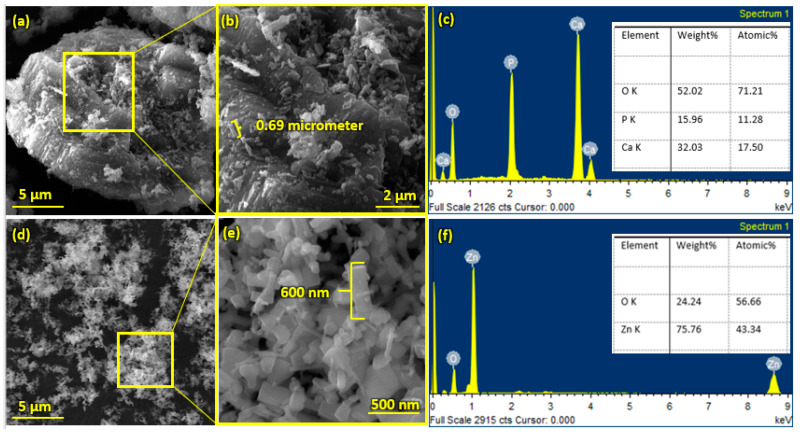
Scanning electron microscopy and EDS of (**a**–**c**) hydroxyapatite (HA) nanopowder and (**d**–**f**) zinc oxide (ZnO) nanoparticles.

**Figure 3:**
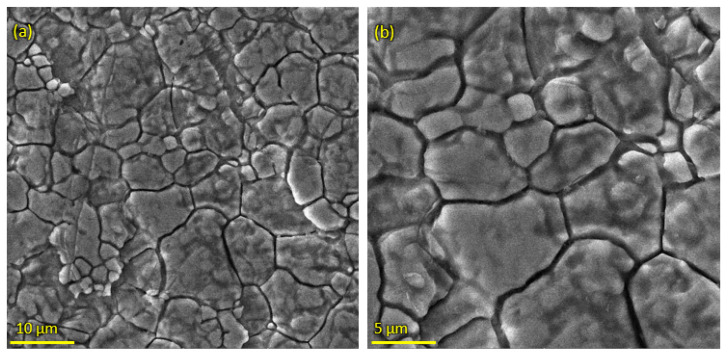
Scanning electron microscopy of SS 316L substrate (**a**) at 10 µm and (**b**) at 5 µm.

**Figure 4:**
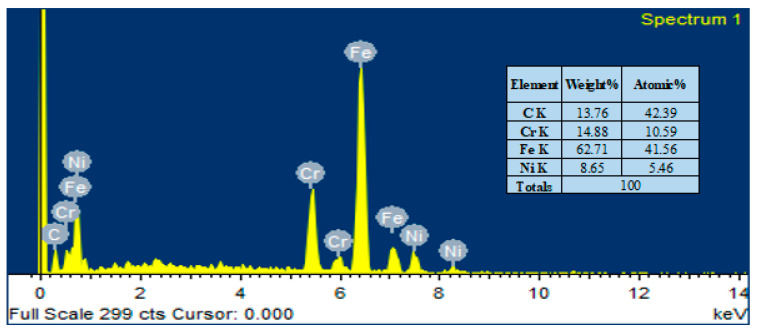
Elemental compositions (EDS) of SS 316L substrate.

**Figure 5:**
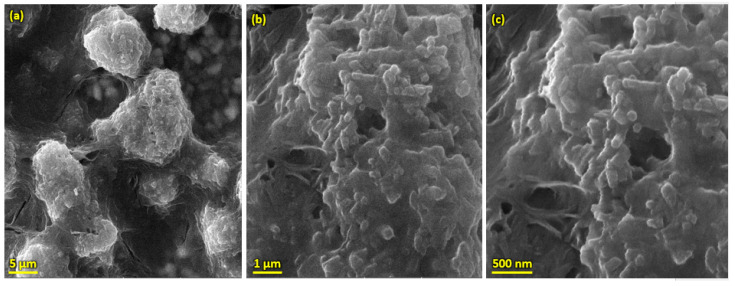
SEM morphology of HA-ZnO coating on SS 316L substrate (**a**) at 5 µm, (**b**) 1 µm and (**c**) 50 nm.

**Figure 6:**
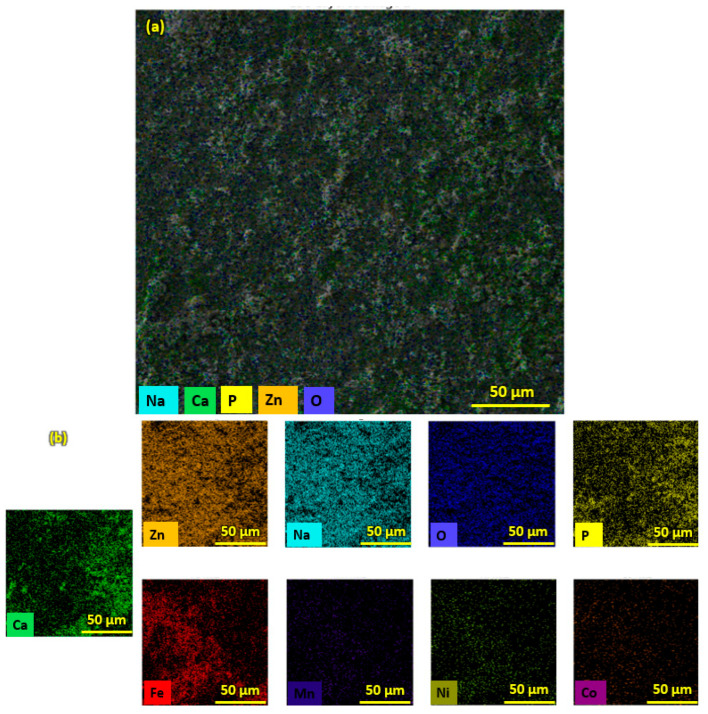
EDS Color maps of (**a**) HA-ZnO coating on SS substrate. (**b**) Elemental compositions of HA-ZnO coating.

Pure HA and ZnO powder was examined using scanning electron microscopy (SEM), as shown in Figure 2. The surface morphology and thickness of the HA-ZnO-coated samples prepared via EPD techniques were examined using SEM. The SEM morphology and EDS of the SS 316L are shown in Figure 3 and Figure 4. The EDS of the SS shows that no impurities existed. The SEM images shows that HA-ZnO coatings were uniform, and no segregation of any particles at any point was detected. The flower-like structure and cluster morphology depicts that the HA and ZnO are properly mixed and are present in the coating, as shown in Figure 5a–c. The images also confirm the proper mixing of HA and ZnO because of the structural distribution among the cluster. Figure 6 represents the color mapping, which leads to the verification of the uniform mixing of the HA powder with the ZnO powder.

**Figure 7:**
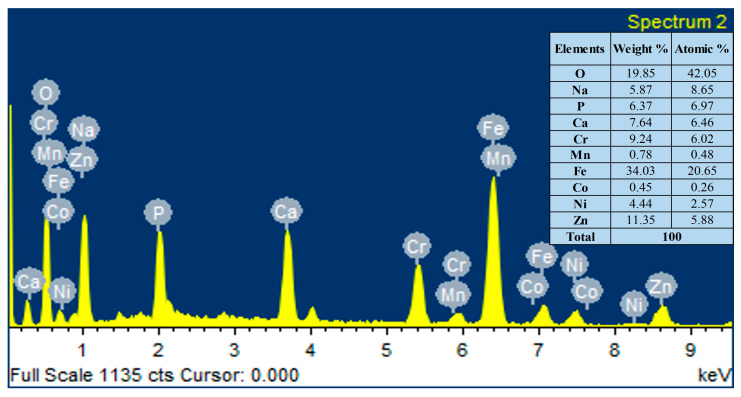
Elemental compositions (EDS) of HA-ZnO coating on SS 316L substrate.

The elemental composition analysis of the HA-ZnO coating applied on the SS substrate was carried out using EDX analysis. The EDX analysis shows the presence of Ca, P, O and ZnO elements, as shown in Figure 7. Therefore, the formation of the HA-ZnO coating is confirmed, in addition to some other elements, i.e., Fe and Cr, which showed the composition of the SS.

**Figure 8:**
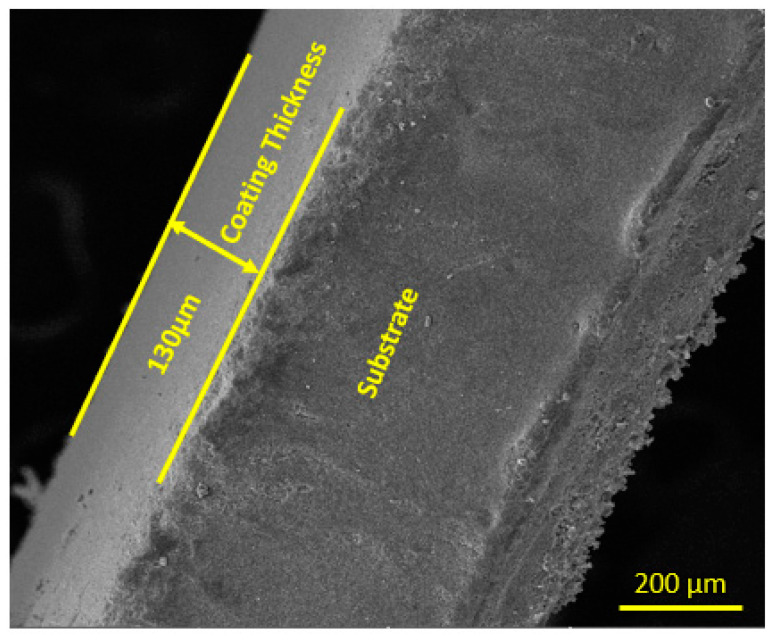
Cross-sectional view and thickness of HA-ZnO coating.

Figure 8 shows that the coating is uniform, compact and has a thickness of about 30 µm. Similar results were achieved by Krause et al. for a Bioglass^®^ coating on a stainless-steel substrate [47].

### 3.2. Surface Roughness Measurement

**Figure 9:**
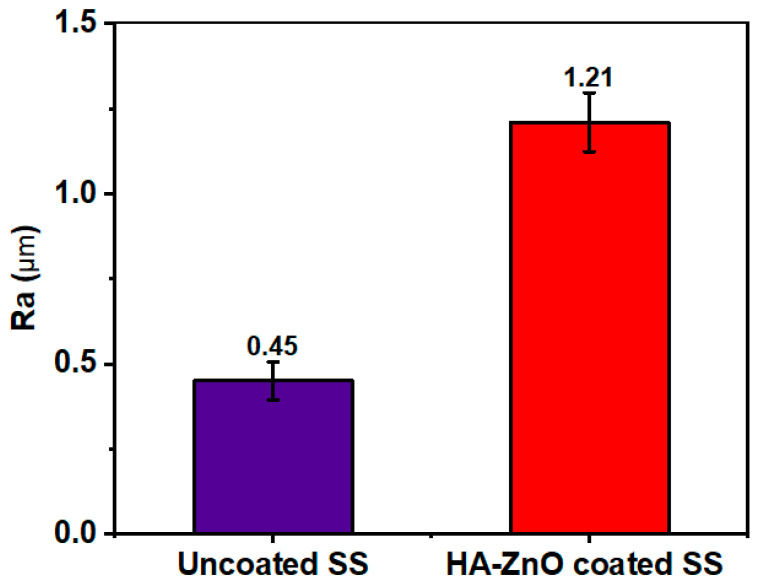
Surface roughness (Ra) of uncoated SS and HA-ZnO coating.

Surface roughness is an important parameter in biomedical materials for physiological activities. Surface roughness is basically cause by the irregularities present on a material’s surface that cause variations in its features. It is characterized by its roughness average (Ra), which measures the surface’s mean deviation from its nominal height over a specific area. Figure 9 shows that the roughness (Ra) of 316L SS is 0.45 µm, while the Ra of HA-ZnO is 1.21 µm, determined using the average of three sample values. Our values for as-deposited coatings lie in the recommended cell roughness range of 1–2 µm [48], and it was shown that the addition of ZnO enhances the surface roughness to the biomedically recommended range [14].

### 3.3. Corrosion Study

**Figure 10:**
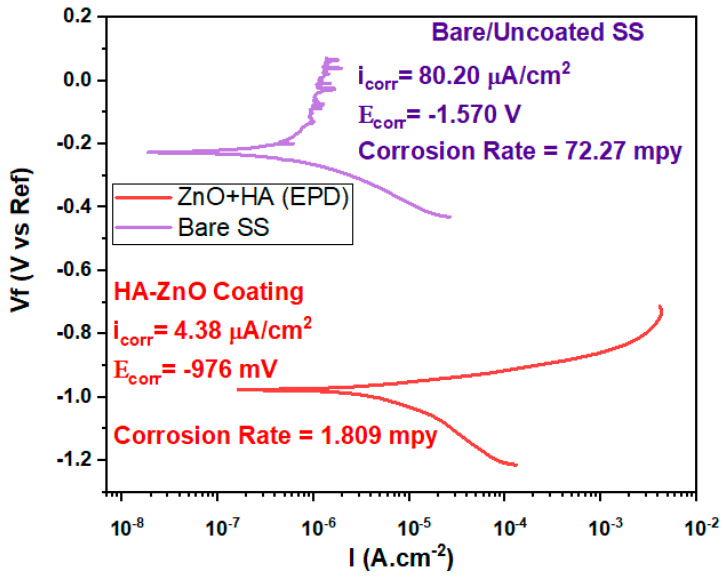
Tafel plot of bare/uncoated SS and HA-ZnO coating in SBF.

Electrochemical corrosion testing was conducted for bare and coated SS substrate samples. Figure 10 shows the potentiodynamic polarization curves for the SS substrate and HA-ZnO-coated samples. The data indicate that an increase in the applied potential results in the passivation of the stainless-steel samples, implying that the formation of a stable chromium oxide layer inhibits further corrosion and slows down the corrosion process. On the contrary, while the applied potential is increased, HA-ZnO coatings did not undergo passivation. When examining the cathodic branch of the polarization curve, it was noted that the use of HA-ZnO coatings led to a significant reduction in cathodic current densities. To determine the corrosion current density, a Tafel fit was applied to the polarization curve, as shown in Figure 10. The corrosion rate of SS was 72.27 mpy, while the HA-ZnO coating successfully decreased the corrosion rate to 1.809 mpy, indicating that the bare SS is more vulnerable to corrosion while the HA-ZnO-coated sample is comparatively good against corrosion resistance in simulated body fluid. Due to the high protection it offers against corrosion, this coating may survive until the healing of the injury. As HA-ZnO usually starts interaction with surrounding tissues within 21 days and a very strong interface is found in-between implant and tissues, this bonding makes the implant durable and long-lasting [49].

### 3.4. FTIR Analysis

**Figure 11:**
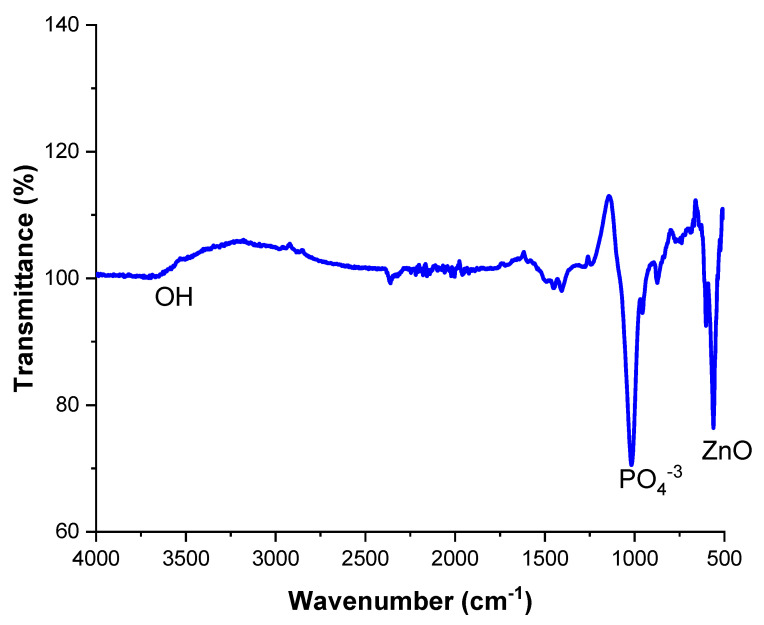
FTIR Spectra of HA-ZnO EPD coating.

Figure 11 shows the results of FITR analysis. The peaks confirm the presence of HA and ZnO compositions in the spectra range of 500–600 and at 3500 cm^−1^. P-O groups are present in the range of 1000–1500 cm^-1^, while OH is in a hydroxyl group detected from HA, detected at a 3600 cm^−1^ wave number. Extra peaks were not examined, which is an indication that no other reaction took place. Ziqi Liu et al. also investigated the same peaks on a double-layer Ca-P sandwiched siloxane composite coating on an Mg alloy for bone tissue applications and reported that the corrosion resistance of the coated samples was improved compared to the Mg alloy substrate [50].

### 3.5. Adhesion Strength

**Figure 12:**
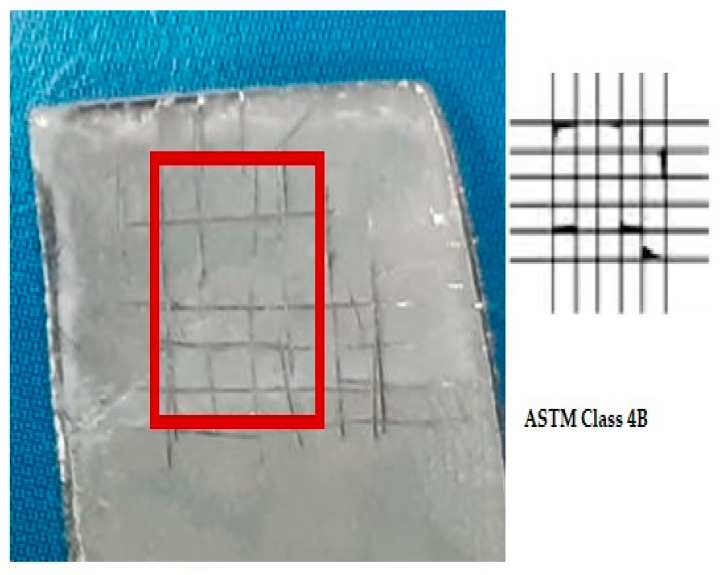
Cross-hatch tape test result of HA-ZnO coating.

To examine the adhesive properties of the coating with the substrate, a cross-hatch tape test was conducted. Figure 12 depicts the results of the cross-hatch tape test for the HA-ZnO composite coatings. The adhesion strength of the EPD coatings was rated as “4B”, according to the ASTM D3359 standard, showing that these coated samples are suitable for ortho-biomedical applications. In addition, it assures that the adhesion rate between the substrate and coating is appropriate and can bear the implantation load. The proper encapsulation and cohesion of HA with ZnO was the reason for the good adhesion with the substrate, as investigated by Mokobia et al. [51]. Ahmadi et al. [52] observed that the addition of ZnO has a positive effect on the cohesive properties of the HA-ZnO coating. This is due to ZnO’s ability to facilitate the synthesis of pure HA crystals while also promoting crystal growth and network formation, resulting in increased strength. ZnO nanoparticles are also useful for enhancing loading efficiency since they possess the same HCP crystal structure as HA. However, when too much ZnO is added, irregularly shaped elongated plates may form in the HA-Z8 coating. HA-Z8 results in the incorporation of much more ZnO NPs. It has been reported that as the thickness of the coating increases, the residual stresses also increase, potentially compromising the overall durability of the coating [52].

### 3.6. XRD Analysis

**Figure 13:**
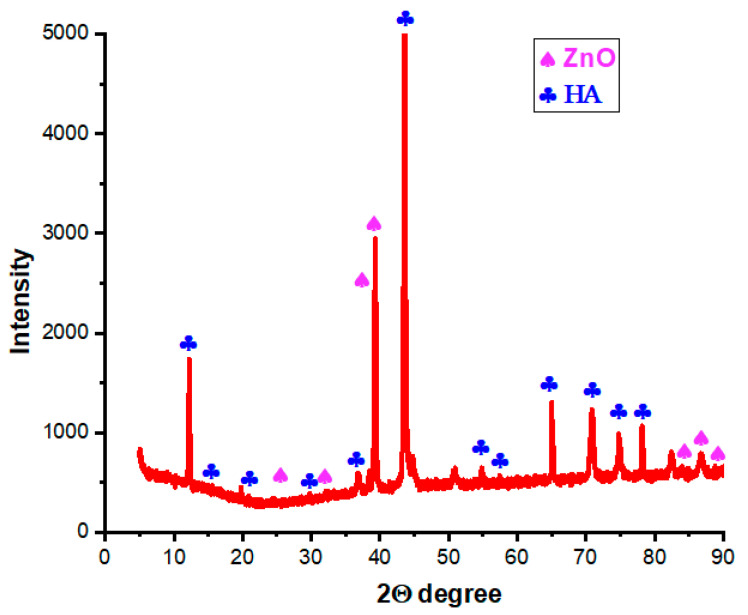
XRD results of HA-ZnO.

Figure 13 shows the XRD patterns of the HA-ZnO composite sample. The results show the compositions of hydroxyapatite and zinc oxide. The broad diffraction peaks are identical to the standard diffraction patterns of ZnO (PDF#21-1486) and HA (PDF#09-0432). The obtained patterns are in agreement with Kim et al. and Heidari et al. [53,54].

### 3.7. In Vitro Bioactivity

In order to check and investigate the in vitro bio-activity, coated samples were immersed in SBF solution. The samples were cut into small pieces to be immersed in the SBF solution. pH and weight were taken into account before and after immersion in SBF. Changes in weight were observed after durations of 24 h, 72 h, 120 h and 168 h. The detailed data of the weight and pH are shown in Table 1. It was observed that after 24 h, the sample lost weight due to adjustment or inflammation factors, as stated by Li. et al. [55]. After 168 h, the sample adjusted to the simulated body fluid and gained weight, and its pH also stabilized. These factors lead towards the interaction of cells withing the bodily fluid.

**Figure 14:**
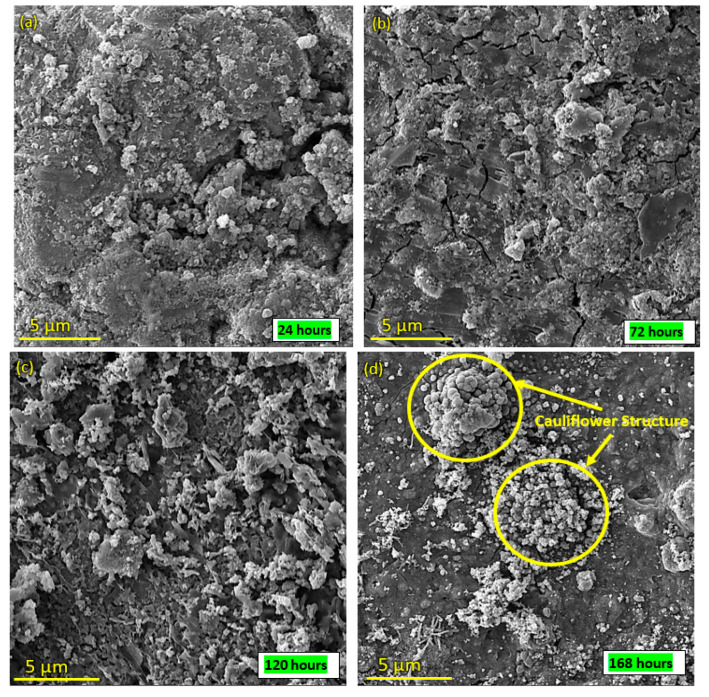
SEM images of HA-ZnO coating after immersion in SBF for (**a**) 24 h; (**b**) 72 h; (**c**) 120 h; (**d**) 168 h.

**Figure 15:**
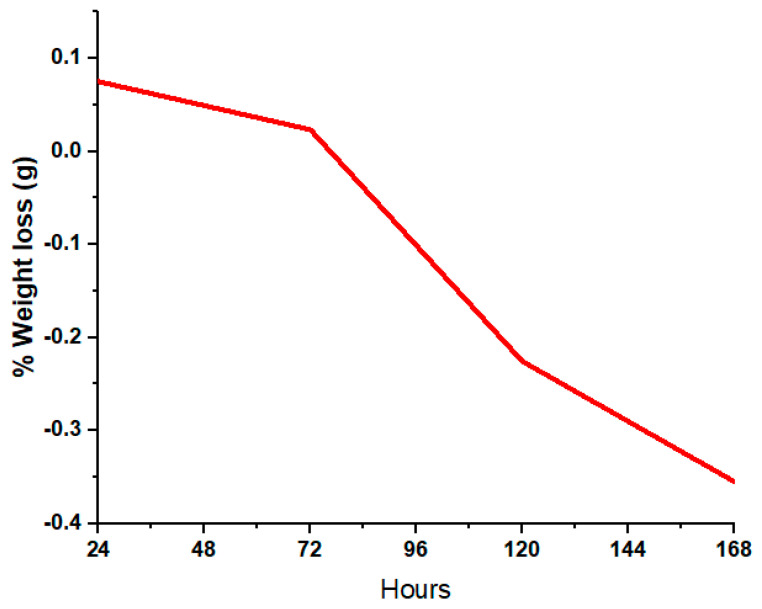
Percentage weight gain of HA-ZnO-coated samples after immersion in SBF.

SEM images of the surface of coated samples were examined during immersion in SBF at different time intervals. Figure 14a–d depict the SEM images after SBF treatment of coated samples for 24 h, 72 h, 120 h and 168 h. Figure 14a indicates that SBF treatment affects the morphology of the coating, as cracks and opening were observed, while Figure 14b–d show that with the passage of time, HA-ZnO shows growth in the form of a cauliflower structure [56], which indicates cell interaction and stability. Figure 15 shows the percentage weight loss of the HA-ZnO-coated samples after immersion in SBF for 24 h, 72 h, 120 h and 168 h.

### 3.8. Cell Viability

**Figure 16:**
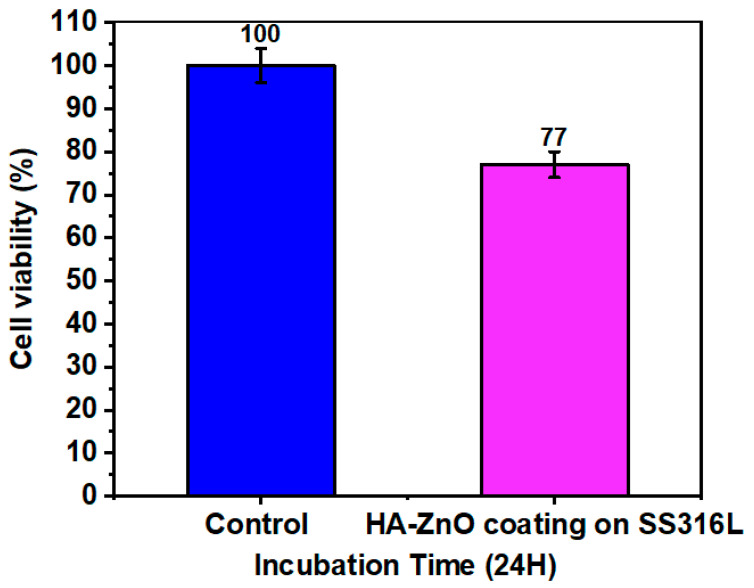
Cell viability of HA-ZnO-coated sample (value = mean ± SD, N = 3).

The viability of the cells on the HA-ZnO coatings compared to the control is depicted in Figure 16. The HA-ZnO layers of the coatings promoted the growth of cells, similar to osteoblasts. The cell density before incubation was 20 × 10^5^. An amount of 50 μL of the cell suspension was added to 50 μL of each sample and incubated for 24 h at 37 °C. After incubation, cells were counted using a hemocytometer, and the obtained cell density was 15.4 × 10^4^, which shows greater than 70% cell viability. Hence, the prepared material is considered to be beneficial for cells. Based on this result, it can be concluded that the cell viability is above 70%, indicating no risk of cytotoxicity. Hence, these coated samples are recommended for use in biomedical implants. Similar results were achieved by T. Alonso et al. [42]. E. Barua et al. also observed the same cell viability in their evaluation of the performance of bone scaffolds made from different hydroxyapatite sources and composed of ZnO-incorporated hydroxyapatite and polymethyl methacrylate tri-components [56]. A. Mehrvarz et al. is in agreement with the cell viability, biocompatibility and antibacterial behavior of electrochemically deposited hydroxyapatite/ZnO porous nanocomposite on a NiTi biomedical alloy [57]. In addition, S. A. Batool is in agreement with the same viability rate for Zn–Mn-doped mesoporous bioactive glass nanoparticle-loaded zein coatings for bioactive and antibacterial orthopedic implants [58].

### 3.9. Antibacterial Activity

**Figure 17:**
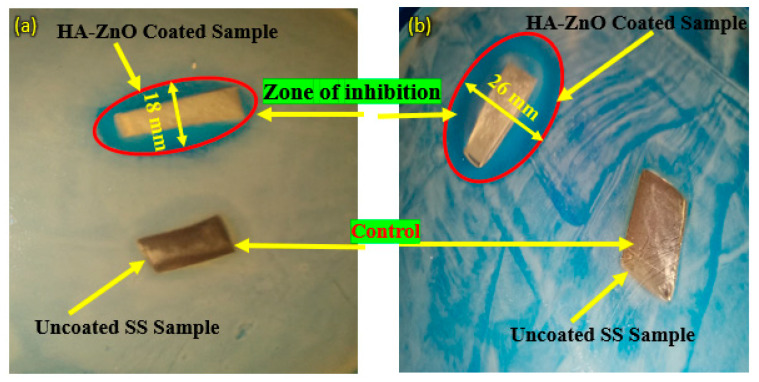
Antibacterial activity of SS 316L and HA-ZnO-coated samples in (**a**) *Staphylococcus aureus* (Gram-positive) medium and (**b**) *Escherichia coli* (Gram-negative) medium.

Figure 17 shows the antibacterial activity of bare/uncoated SS and HA-ZnO-coated samples. The figure shows that the *Staphylococcus aureus* (Gram-positive) and *Escherichia coli* (Gram-negative) bacteria attacked the bare sample while the coated sample was protected from bacterial attack, as also mentioned by Venezia at al. for HA-zinc oxide [59]. In the Gram-negative sample, the coated sample was protected and a 26 mm inhibition zone was achieved, while in the Gram-positive bacterial attack, the zone of inhibition was found to be 18 mm. In contrast to this, both bare/uncoated SS samples were observed to be under bacterial attack. The same results were also investigated for ZnO by V. Venezia et al. [59]. Similarly, K. Madhusudana Rao [16] observed that the cell wall of *E. coli* is generally thin and made up of peptidoglycan, which makes it less susceptible to reactive oxygen species (ROS) generated from ZnO. Reactive oxygen species (ROS) are chemically reactive molecules that contain oxygen, such as superoxide, hydrogen peroxide and hydroxyl radicals [60]. On the other hand, *S. aureus* has multiple layers of peptidoglycan with numerous pores, making it more vulnerable to ROS and leading to cell disruption [61]. Therefore, ZnO nanoparticles have been found to exhibit greater antibacterial activity against *S. aureus* than *E. coli*. HA-ZnO NCH hydrogels displayed more potent antibacterial activity against *E. coli* than *S. aureus* [16].

## 4. Conclusions

In this paper, we have studied the morphology, roughness, corrosion resistance and adhesion properties of HA-ZnO coatings deposited on an SS 316 substrate via EPD, revealing the in vitro and antibacterial performance of such coatings. The resultant coatings appeared to be uniform and evenly distributed, the porous structure of HA being filled with ZnO. The coating’s roughness was improved due to the cohesiveness and surface adoptability of ZnO, making it suitable for use in an SBF environment. The observed improvements in adhesion and inhibition zone collectively reflect the suitability of HA-ZnO coatings prepared via EPD for bio-implant applications.

## Figures and Tables

**Table 1:** HA-ZnO nanocomposite-coated samples after immersion in SBF.

Time (h)	Initial pH	Final pH	Change in pH	Initial Weight (g)	Final Weight (g)	Change in Weight (g)
24	7.4	7.32	0.08	0.4001	0.3998	−0.0003
72	7.4	7.35	0.05	0.4292	0.4291	0.0008
120	7.4	7.38	0.02	0.4437	0.4447	0.001
168	7.4	7.4	0	0.4231	0.4246	0.0014

## Data Availability

Not applicable.

## Supplementary Materials

The following supporting information can be downloaded at: https://www.mdpi.com/article/10.3390/bioengineering10060693/s1: Figure S1. Experimental set-up for electrophoretic deposition of HA-ZnO on SS 316L; Figure S2. HA-ZnO Coated on SS 316L Substrate.

## Sample Availability

All samples were consumed.

## References

[1] Wright M., Uddin A. (2012). Organic—inorganic hybrid solar cells: A comparative review. Sol. Energy Mater. Sol. Cells.

[2] Lacefield W. (1988). Hydroxyapatite coatings. Ann. N. Y. Acad. Sci..

[3] Schatkoski V.M., do Amaral Montanheiro T.L., de Menezes B.R.C., Pereira R.M., Rodrigues K.F., Ribas R.G., da Silva D.M., Thim G.P. (2021). Current advances concerning the most cited metal ions doped bioceramics and silicate-based bioactive glasses for bone tissue engineering. Ceram. Int..

[4] Cheang P., Khor K. (1996). Addressing processing problems associated with plasma spraying of hydroxyapatite coatings. Biomaterials.

[5] Gibson I.R., Ke S., Best S., Bonfield W. (2001). Effect of powder characteristics on the sinterability of hydroxyapatite powders. J. Mater. Sci. Mater. Med..

[6] Wang Y., Khor K., Cheang P. (1998). Thermal spraying of functionally graded calcium phosphate coatings for biomedical implants. J. Therm. Spray Technol..

[7] Kweh S., Khor K., Cheang P. (2000). Plasma-sprayed hydroxyapatite (HA) coatings with flame-spheroidized feedstock: Microstructure and mechanical properties. Biomaterials.

[8] Liu Y.-C., Lee Y.-T., Huang T.-C., Lin G.-S., Chen Y.-W., Lee B.-S., Tung K.-L. (2021). In vitro bioactivity and antibacterial activity of strontium-, magnesium-, and zinc-multidoped hydroxyapatite porous coatings applied via atmospheric plasma spraying. ACS Appl. Bio Mater..

[9] Goldmann W.H. (2021). Biosensitive and antibacterial coatings on metallic material for medical applications. Cell Biol. Int..

[10] Sabzi M., Far S.M., Dezfuli S.M. (2018). Characterization of bioactivity behavior and corrosion responses of hydroxyapatite-ZnO nanostructured coating deposited on NiTi shape memory alloy. Ceram. Int..

[11] Kand A.J.T., Afaghi F., Tabrizi A.T., Aghajani H., Kivrak H.D. (2021). Electrochemical evaluation of the hydroxyapatite coating synthesized on the AZ91 by electrophoretic deposition route. Synth. Sinter..

[12] Stanković A., Dimitrijević S., Uskoković D. (2013). Influence of size scale and morphology on antibacterial properties of ZnO powders hydrothemally synthesized using different surface stabilizing agents. Colloids Surf. B Biointerfaces.

[13] Sharma A., Ranjit R., Kumar N., Kumar M., Giri B.S. (2023). Nanoparticles based nanosensors: Principles and their applications in active packaging for food quality and safety detection. Biochem. Eng. J..

[14] Bansal P., Singh G., Sidhu H.S. (2020). Investigation of surface properties and corrosion behavior of plasma sprayed HA/ZnO coatings prepared on AZ31 Mg alloy. Surf. Coat. Technol..

[15] Turlybekuly A., Pogrebnjak A., Sukhodub L., Sukhodub L.B., Kistaubayeva A., Savitskaya I., Shokatayeva D., Bondar O.V., Shaimardanov Z.K., Plotnikov S.V. (2019). Synthesis, characterization, in vitro biocompatibility and antibacterial properties study of nanocomposite materials based on hydroxyapatite-biphasic ZnO micro-and nanoparticles embedded in Alginate matrix. Mater. Sci. Eng. C.

[16] Rao K.M., Suneetha M., Zo S., Duck K.H., Han S.S. (2019). One-pot synthesis of ZnO nanobelt-like structures in hyaluronan hydrogels for wound dressing applications. Carbohydr. Polym..

[17] Maimaiti B., Zhang N., Yan L., Luo J., Xie C., Wang Y., Ma C., Ye T. (2020). Stable ZnO-doped hydroxyapatite nanocoating for anti-infection and osteogenic on titanium. Colloids Surf. B Biointerfaces.

[18] Bansal P., Upadhyay L. (2016). Effect of turning parameters on tool wear, surface roughness and metal removal rate of alumina reinforced aluminum composite. Procedia Technol..

[19] Zhou J., Li K., Wang B., Ai F. (2020). Nano-hydroxyapatite/ZnO coating prepared on a biodegradable Mg–Zn–Ca bulk metallic glass by one-step hydrothermal method in acid situation. Ceram. Int..

[20] Akram W., Farhan Rafique A., Maqsood N., Khan A., Badshah S., Khan R.U. (2020). Characterization of PTFE film on 316L stainless steel deposited through spin coating and its anticorrosion performance in multi acidic mediums. Materials.

[21] Ahmed Y., Yasir M., Ur Rehman M.A. (2020). Fabrication and characterization of zein/hydroxyapatite composite coatings for biomedical applications. Surfaces.

[22] Ali S., Abdul Rani A.M., Mufti R.A., Azam F.I., Hastuty S., Baig Z., Hussain M., Shehzad N. (2019). The influence of nitrogen absorption on microstructure, properties and cytotoxicity assessment of 316l stainless steel alloy reinforced with boron and niobium. Processes.

[23] Liu J., Wang Y., Ma J., Peng Y., Wang A. (2019). A review on bidirectional analogies between the photocatalysis and antibacterial properties of ZnO. J. Alloys Compd..

[24] Varshney S., Nigam A., Singh A., Samanta S.K., Mishra N., Tewari R. (2022). Antibacterial, structural, and mechanical properties of MgO/ZnO nanocomposites and its HA-based bio-ceramics; synthesized via physio-chemical route for biomedical applications. Mater. Technol..

[25] Radin S., Ducheyne P. (2007). Controlled release of vancomycin from thin sol–gel films on titanium alloy fracture plate material. Biomaterials.

[26] Edupuganti O.P., Antoci V., King S.B., Jose B., Adams C.S., Parvizi J., Shapiro I.M., Zeiger A.R., Hickok N.J., Wickstrom E. (2007). Covalent bonding of vancomycin to Ti6Al4V alloy pins provides long-term inhibition of *Staphylococcus aureus* colonization. Bioorganic Med. Chem. Lett..

[27] Teker D., Muhaffel F., Menekse M., Karaguler N.G., Baydogan M., Cimenoglu H. (2015). Characteristics of multi-layer coating formed on commercially pure titanium for biomedical applications. Mater. Sci. Eng. C.

[28] Chen W., Liu Y., Courtney H., Bettenga M., Agrawal C., Bumgardner J., Ong J. (2006). In vitro anti-bacterial and biological properties of magnetron co-sputtered silver-containing hydroxyapatite coating. Biomaterials.

[29] Jeon H.-J., Yi S.-C., Oh S.-G. (2003). Preparation and antibacterial effects of Ag–SiO_2_ thin films by sol–gel method. Biomaterials.

[30] Ewald A., Glückermann S.K., Thull R., Gbureck U. (2006). Antimicrobial titanium/silver PVD coatings on titanium. Biomed. Eng. Online.

[31] Seuss S., Lehmann M., Boccaccini A.R. (2014). Alternating current electrophoretic deposition of antibacterial bioactive glass-chitosan composite coatings. Int. J. Mol. Sci..

[32] Raza M.A., Ali A., Ghauri F.A., Aslam A., Yaqoob K., Wasay A., Raffi M. (2017). Electrochemical behavior of graphene coatings deposited on copper metal by electrophoretic deposition and chemical vapor deposition. Surf. Coat. Technol..

[33] Acheson J.G., Gallagher E., Ward J., McKillop S., FitzGibbon B., Boyd A., Meenan B., Lemoine P., McGarry J. (2022). Shear testing and failure modelling of calcium phosphate coated AZ31 magnesium alloys for orthopaedic applications. Surf. Coat. Technol..

[34] Jaafar A., Hecker C., Árki P., Joseph Y. (2020). Sol-gel derived hydroxyapatite coatings for titanium implants: A review. Bioengineering.

[35] Pradeep D., Venkatesh C., Nithin H. (2022). Review on tribological and mechanical behavior in HVOF thermal-sprayed composite coatings. J. Bio-Tribo-Corros..

[36] Fotovvati B., Namdari N., Dehghanghadikolaei A. (2019). On coating techniques for surface protection: A review. J. Manuf. Mater. Process..

[37] Li T.-T., Ling L., Lin M.-C., Peng H.-K., Ren H.-T., Lou C.-W., Lin J.-H. (2020). Recent advances in multifunctional hydroxyapatite coating by electrochemical deposition. J. Mater. Sci..

[38] Saadati A., Khiarak B.N., Zahraei A.A., Nourbakhsh A., Mohammadzadeh H. (2021). Electrochemical characterization of electrophoretically deposited hydroxyapatite/chitosan/graphene oxide composite coating on Mg substrate. Surf. Interfaces.

[39] Hanif M.B., Gao J.-T., Shaheen K., Wang Y.-P., Yasir M., Li C.-J., Li C.-X. (2021). Microstructural analysis of highly active cathode material La0. 7Sr0. 3Ti0. 15Fe0. 65Ni0. 2O3-δ (LSTFN) by optimizing different processing parameters. Ceram. Int..

[40] Damiano M. (2020). Surface Profile: Research and Insight. J. Prot. Coat. Linings.

[41] Ahmed U., Yi L., Fei L.F., Yasir M., Li C.-J., Li C.-X. (2021). Enhancement of corrosion resistance and tribological properties of LA43M Mg alloy by cold-sprayed aluminum coatings reinforced with alumina and carbon nanotubes. J. Therm. Spray Technol..

[42] Oyane A., Kim H.M., Furuya T., Kokubo T., Miyazaki T., Nakamura T. (2003). Preparation and assessment of revised simulated body fluids. J. Biomed. Mater. Res. Part A Off. J. Soc. Biomater. Jpn. Soc. Biomater. Aust. Soc. Biomater. Korean Soc. Biomater..

[43] Shaltout A.A., Allam M.A., Moharram M.A. (2011). FTIR spectroscopic, thermal and XRD characterization of hydroxyapatite from new natural sources. Spectrochim. Acta Part A Mol. Biomol. Spectrosc..

[44] (2003). Standard Test Methods for Measuring Adhesion by Tape Test.

[45] Kokubo T., Takadama H. (2006). How useful is SBF in predicting in vivo bone bioactivity?. Biomaterials.

[46] Sayed M.A., El-Rahman T.M.A., Abdelsalam H., Ali A.M., Hamdy M.M., Badr Y.A., El Rahman N.H.A., El-Latif S.M.A., Mostafa S.H., Mohamed S.S. (2022). Attractive study of the antimicrobial, antiviral, and cytotoxic activity of novel synthesized silver chromite nanocomposites. BMC Chem..

[47] Krause D., Thomas B., Leinenbach C., Eifler D., Minay E.J., Boccaccini A.R. (2006). The electrophoretic deposition of Bioglass^®^ particles on stainless steel and Nitinol substrates. Surf. Coat. Technol..

[48] Huang C.-L., Huang K.-T., Lee T.-M. (2023). The biological responses of osteoblasts on titanium: Effect of oxygen level and surface roughness. J. Formos. Med. Assoc..

[49] Zuhdi S.A. (2023). Analysis of Mechanical Properties of Biodegradable Implants Material: Pure Zinc and Stainless Steel 316L for Bone External Fixation.

[50] Liu Z., Wang T., Xu Y., Liang C., Li G., Guo Y., Zhang Z., Lian J., Ren L. (2023). Double-layer calcium phosphate sandwiched siloxane composite coating to enhance corrosion resistance and biocompatibility of magnesium alloys for bone tissue engineering. Prog. Org. Coat..

[51] Mokobia K.E., Ifijen I.H., Ikhuoria E.U. (2023). ZnO-NPs-coated implants with osteogenic properties for enhanced osseointegration. TMS 2023 152nd Annual Meeting & Exhibition Supplemental Proceedings.

[52] Ahmadi R., Afshar A. (2021). In vitro study: Bond strength, electrochemical and biocompatibility evaluations of TiO_2_/Al_2_O_3_ reinforced hydroxyapatite sol–gel coatings on 316L SS. Surf. Coat. Technol..

[53] Heidari F., Bahrololoom M.E., Vashaee D., Tayebi L. (2015). In situ preparation of iron oxide nanoparticles in natural hydroxyapatite/chitosan matrix for bone tissue engineering application. Ceram. Int..

[54] Heidari F., Razavi M., Bahrololoom M.E., Bazargan-Lari R., Vashaee D., Kotturi H., Tayebi L. (2016). Mechanical properties of natural chitosan/hydroxyapatite/magnetite nanocomposites for tissue engineering applications. Mater. Sci. Eng. C.

[55] Li Y., Zhou M., Zheng W., Yang J., Jiang N. (2023). Scaffold-based tissue engineering strategies for soft–hard interface regeneration. Regen. Biomater..

[56] Barua E., Das A., Deoghare A.B., Pamu D., Deb P., Lala S.D., Chatterjee S. (2023). Performance of ZnO-Incorporated Hydroxyapatite/Polymethyl Methacrylate Tri-Component Composite Bone Scaffolds Fabricated from Varying Sources of Hydroxyapatite. J. Mater. Eng. Perform..

[57] Mehrvarz A., Khalil-Allafi J., Khosrowshahi A.K. (2022). Biocompatibility and antibacterial behavior of electrochemically deposited Hydroxyapatite/ZnO porous nanocomposite on NiTi biomedical alloy. Ceram. Int..

[58] Batool S.A., Ahmad K., Irfan M., Ur Rehman M.A. (2022). Zn–Mn-Doped Mesoporous Bioactive Glass Nanoparticle-Loaded Zein Coatings for Bioactive and Antibacterial Orthopedic Implants. J. Funct. Biomater..

[59] Venezia V., Verrillo M., Gallucci N., Di Girolamo R., Luciani G., D’Errico G., Paduano L., Piccolo A., Vitiello G. (2023). Exploiting bioderived humic acids: A molecular combination with ZnO nanoparticles leads to nanostructured hybrid interfaces with enhanced pro-oxidant and antibacterial activity. J. Environ. Chem. Eng..

[60] Biswas A., Kar U., Jana N.R. (2022). Cytotoxicity of ZnO nanoparticles under dark conditions via oxygen vacancy dependent reactive oxygen species generation. Phys. Chem. Chem. Phys..

[61] Cheung G.Y., Bae J.S., Otto M. (2021). Pathogenicity and virulence of *Staphylococcus aureus*. Virulence.

