# Supplementary material for: Enhancement of Antibacterial Properties, Surface Morphology and In Vitro Bioactivity of Hydroxyapatite-Zinc Oxide Nanocomposite Coating by Electrophoretic Deposition Technique
Source: Bioengineering (Basel). 2023 Jun 7;10(6):693. doi: 10.3390/bioengineering10060693 (PMC10295605; doi:10.3390/bioengineering10060693)
Supplement: Supplementary file 1 [file bioengineering-10-00693-s001.zip › bioengineering-2317236-supplementary.pdf]

Supplementary Materials:

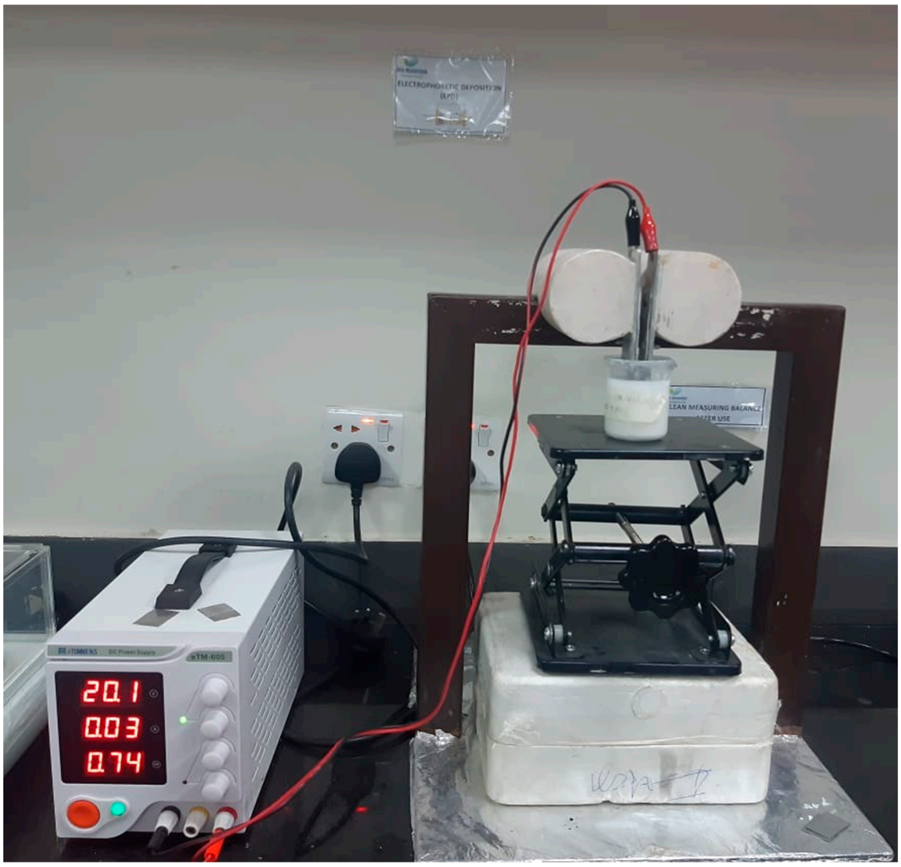

Figure S1. Experimental set-up for electrophoretic deposition of HA-ZnO on SS 316L.

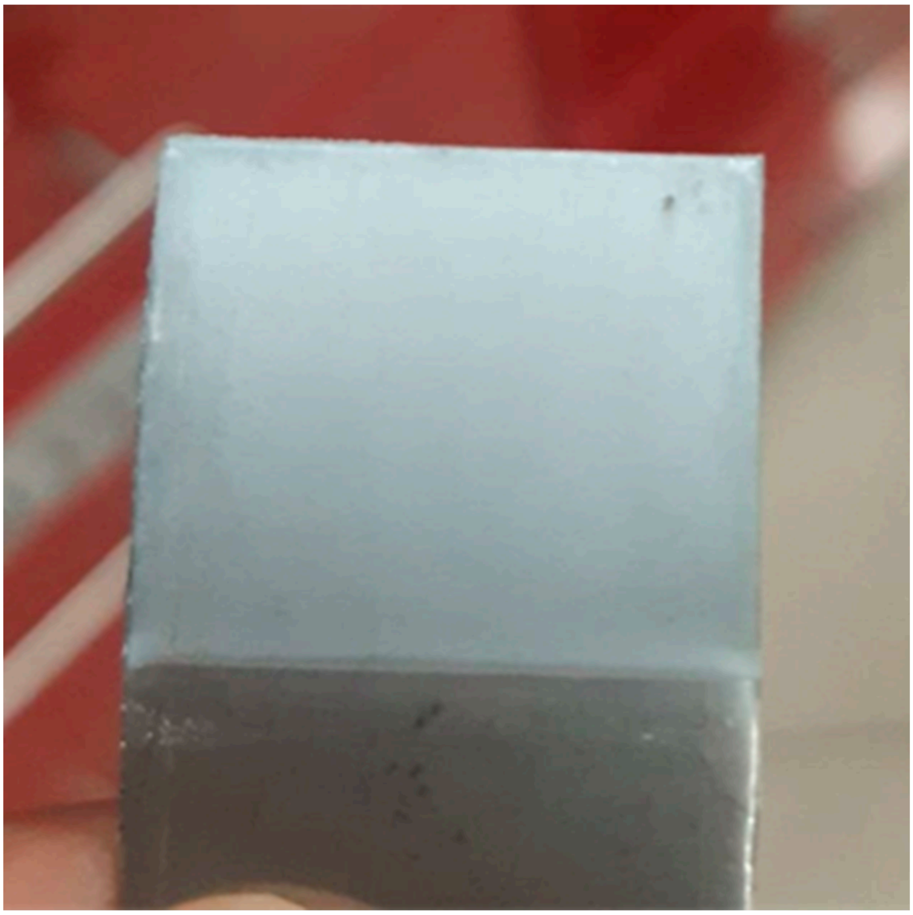

Figure S2. HA-ZnO Coated on SS 316L Substrate.
